# Letter to: neurological manifestation of coeliac disease: many immunological markers suggesting a further expression of gluten-induced secondary autoimmunity 

**Published:** 2021

**Authors:** Markus Wilhelmi

**Affiliations:** *Medical Clinic I, Gastroenterology and Hepatology, Nutritional Medicine University Hospital, Frankfurt, Germany*

**To The Editor**

 I read with great interest the very interesting review article by Osman et al who addressed the issue of neurological manifestations of coeliac disease (CD), with particular emphasis on gluten ataxia and immunological injury (1). 

They have reviewed in detail the most relevant studies addressing the relationship of CD with cerebellar ataxia (CA) and immunopathology of enteric CD in comparison to neuronal pathology. The role of gluten-free diet (GFD) with special emphasis on its effect on improving symptoms of ataxia was also reviewed.

This topic is of great relevance since neurological manifestations can occur in about 10% of CD patients with CA, peripheral neuropathy, seizures, headache, cognitive impartment and various neuropsychiatrist disorders representing potentially extraintestinal manifestations of CD, as properly stated by the authors.

Although I consider this review to be of significant interest and very up-to-date, I feel that some issues would have deserved more emphasis owing to their clinical relevance.

In particular, when the Author discussed the potential spectrum of neurological manifestations occurring in CD patients, they omitted to mention multiple sclerosis, multifocal leukoencephalopathy, chorea, and attention/memory impairment, all of which are well known to be other neurological disorders potentially associated to CD (2). 

Of major interest, I would also like to point out that among the autoantibodies potentially detectable in CD patients with neurological manifestations, other than anti-ganglioside antibodies, it is worth mentioning also anti-neuronal antibodies to central/enteric nervous systems detected by indirect immunofluorescence as previously reported (3-5).

These antibodies can be considered markers of neurological manifestations as they have been reported in more than 60% of CD patients with neurological manifestations and therefore should be included in the immunological assessment of CD patients due to their major clinical significance.

## Conflict of interests

The authors declare that they have no conflict of interest.
