# Supplementary material for: Letter to: neurological manifestation of coeliac disease: many immunological markers suggesting a further expression of gluten-induced secondary autoimmunity
Source: Gastroenterol Hepatol Bed Bench. 2021 Spring;14(2):193–4. (PMC8101522)
Supplement: Related texts

# Reply: RE to Markus Wilhelmi letter

**Published:** 2021

**Authors:** Dina Osman, Seemeen Umar, Humayun Muhammad, Esmaeil Nikfekr, Kamran Rostami, Sauid Ishaq

**Affiliations:** 1 * North Cumbria University Hospital NHSF Trust, UK*; 2 * York Teaching Hospital, NHSF Trust, UK *; 3 * University of Roehampton, London, UK*; 4 * Nottingham University Hospitals, Queen's Medical Centre, UK *; 5 * Department of Gastroenterology Mid-Central District Health Board, Palmerston North, New Zealand *; 6 * Dudley Group of Hospitals NHSF Trust, UK*

**To The Editor**

Thanks for your recent letter and your interest in our review article (1). We read your encouraging remarks about the review and thanks you for that.

You have raised issues about the clinical spectrum of the neurological involvement. We feel this was an omission and although the article was mainly concentrating on the GA, some degree of discussion or a table would have been beneficial to encompass this area of neurological involvement of the CD. This, however, may be noted there are word limits which also needs consideration and at times it is difficult to accommodate all details. Your other comment was valuable and anti-neuronal antibodies should have been included.

## References

[1] Osman D, Umar S, Muhammad H, Nikfekr E, Rostami K, Ishaq S (2021). Neurological manifestation of coeliac disease with particular emphasis on gluten ataxia and immunological injury: a review article. Gastroenterol Hepatol Bed Bench.
